# Supplementary material for: The successor representation subserves hierarchical abstraction for goal-directed behavior
Source: PLoS Comput Biol. 2024 Feb 20;20(2):e1011312. doi: 10.1371/journal.pcbi.1011312 (PMC10906840; doi:10.1371/journal.pcbi.1011312)
Supplement: Attachment — Submitted filename: Response to reviewers.docx [file pcbi.1011312.s003.docx]

**Submission PLOS computational biology (PCOMPBIOL-D-23-01033)**

**Response to reviewers**

Dear Dr. Beierholm, Dr. Graham,

Thank you for considering our manuscript “the successor representation subserves hierarchical abstraction for goal-directed behavior” for a revised submission at your journal. We highly appreciate the careful attention to the paper spent by you and the reviewers. We have modified the manuscript in line with your suggestions and believe that the manuscript has much improved in so doing. All the changes are highlighted in the manuscript. Please find below a point-by-point response to the comments made by the reviewers, which we hope to have addressed adequately. Any references included in the responses we make to the reviewer comments can be found in the reference list of the main manuscript.

Besides modifications in response to the reviewers, we fixed a notational error in the application of the Gram-Schmidt process for successively orthogonalizing the regressors (lines 1114-1118). Several grammatical and linguistic errors were fixed, and a few references have been added, which are highlighted through tracked changes in the manuscript as well.

We look forward to hearing from you in due time regarding our submission and to respond to any further questions and comments you may have.

Sincerely,

Sven Wientjes and Clay B. Holroyd

**Reviewer 1**

**R1.1** *The authors refer to “hierarchical” structure throughout the paper (and even in the title). In their experiments, they consider an environment with two levels of structure: the fine-scale structure of the connections between rooms and the large-scale structure of the wings (or tightly connected groups of rooms). Traditionally, this sort of two-level structure is referred to as “community” structure, as the authors correctly point out throughout the paper. In fact, this exact graph structure has been studied previously, always (to my knowledge) in the context of community structure. Hierarchical structure, by contrast, typically refers to nested communities with at least 3 levels of description (see a recent preprint on learning hierarchical structure:* [*https://arxiv.org/abs/2309.02665*](https://arxiv.org/abs/2309.02665)*). While this is simply a matter of semantics (not a critique of the research itself), in keeping with previous work this paper would be more accurately described as a study of community structure learning rather than hierarchical structure.*

- We thank the reviewer for this clarification. We agree that the manuscript was written confusingly with respect to the dissociation between learning the community structure of a graph (e.g. using a successor representation), and deriving a hierarchical abstraction from this (e.g. represent the communities as three different abstract states). That said, we would argue that only the latter constitutes true hierarchical abstraction, and note that this terminology is frequently used within the literature on hierarchical reinforcement learning to address exactly this type of abstraction. When it comes to learning community structure, explicit abstraction is not necessary, as shown for example by the free energy – mental error model proposed previously (Lynn et al., 2020). In an attempt to clarify this distinction, we have changed the term “hierarchical” when referring to statistical learning of community structure (e.g. successor representation learning), to learning “higher-order structure” of a graph, which is not necessarily hierarchical but does encompass community structure. What follows is an overview of phrases that changed to accommodate this clarification:
- Line 23-24: Previous research has shown that humans are sensitive to a form of higher-order statistical learning named “community structure”.
- Line 78-79: Additionally, the successor representation can explain how people segment events based on the graph topology of the events, capturing higher-order structure in the environment (29–31).
- Line 280-282: This effect has been observed in previous experiments and has been defined as a signature of higher-order predictive learning (29,40,41).
- Line 771-775: Specific evidence exists that humans can learn predictive representations that are sensitive to higher-order aspects of tasks (29,30,40,41). As far as we are aware, this study is the first to show that individual differences in higher-order predictive learning ability predict individual differences in hierarchical abstraction during goal-directed decision making.
- Line 776-778: In order to link predictive representation learning to hierarchically-informed decision making, we adapted a task that has been previously leveraged to show higher-order influences on predictive learning (30).

**R1.2** *When defining the transition structure between rooms, the authors choose a graph with some directed transitions that are not allowed while their reverse occur twice as often as all other transitions (Fig. 1C). However, this choice is likely to have undesirable consequences for the behavior of the participants. For example, having some transitions that occur twice as often as the rest is likely to lead to differences in reaction times (which the authors then have to account for in their regressions). These differences in transitions then give rise to differences between nodes. For example, node 3 has four different incoming and outgoing transitions, while nodes 1, 2, 4, and 5 only have three different incoming and outgoing transitions. I believe the authors could have avoided these issues with a different choice of allowed transitions. For example, in Fig. 1Bii the authors could have allowed directed transitions (1,2), (1,3), (2,3), (2,4), (3,4), (3,5), (4,1), (4,5), (5,2), and (5,6). Then the transitions for the other response would just be the direct reverse. Can the authors explain why they don’t choose transitions such as these, which preserve the symmetries between different connections and different nodes? If the authors have a justification, this should be included in the main text.*

- We thank the reviewer for this insightful suggestion. We have simulated exactly the action-outcome mapping proposed by the reviewer. We conclude the action-outcome mapping proposed by the reviewer works against dissociating influences of model-based (or successor representation) decision making from influences of hierarchical abstraction on decision making. The following text was added to S1 Appendix (section “a symmetric action-outcome mapping”) elaborating on this (see S1 Appendix for relevant Fig. S13):

An inelegant aspect of our task design is the removal of certain edges and the duplication of others across both available actions. It is in fact possible to design action-outcome mappings that do not remove or duplicate edges (e.g. Fig. S13A), and yields desired node visitation statistics under both a random walk and a one-directional policy (Fig. S13B). However, when comparing an optimal model-based policy with the explicit hierarchical policy, we can see there is only one room in each wing where the predictions from these models consistently differ (Fig. S13C). In the figure this would correspond to room 2 when taking action i, and room 4 when taking action ii (Fig. S13A). Crucially, the room where this “antirotation” becomes optimal is directly reached by transitioning away from the boundary room connecting to the goal community, when following the rotational action. For example, when in room 1 and taking action i, it is possible to transition to room 4. This is the room where the antirotation is optimal, and the participant should switch to action ii (which has a 50% chance of returning to room 1). However, they might do so more because they identified a failure to make the desired transition into the goal community, instead of having learned that specifically room 4 allows for a more optimal antirotational action. Similarly, if participants learn the heuristic that actions are reversible, they might reverse their action to reach this boundary state again, even though they did not actually learn a detailed model of the museum layout. This could be overcome by excluding trials where room 4 was preceded by room 1 (i.e., exclude trials where the room with an optimal anti-rotation was preceded by a room at the boundary connecting to the goal community). However, this would come at the cost of statistical power, as it is likely 50% (and even more if participants behave optimally) of trials where the room with an optimal anti-rotation is reached following the boundary room connecting to the goal community. Notably, any action-outcome mapping that would i) maintain all transitions of the original graph as shown in main text Fig. 1A, and ii) not allow for reciprocal transitions given the same action, would display this potential confound. Crucially, participants’ sensitivity to lower-level task structure as revealed in main text Fig. 2G are not subject to these limitations given the balanced action-outcome mapping.

- We do note that some readers might prefer reciprocal transitions between nodes given the same action, as opposed to the solution we went for (removing and duplicating some transitions). A good mapping could have been one where Fig. 1Bii would allow for (1,3), (1,4), (2,3), (2,5), (3,4), (3,5), (4,1), (4,2), (5,2), (5,6) and then Fig. 1Bi would allow for (1,15), (1,2), (2,1), (2,4), (3,1), (3,2), (4,3), (4,5), (5,3), (5,4). However, we posit that allowing participants to return to the previous room upon selecting the same action is counterintuitive, as the pertinent hierarchical abstraction subtly steers an interpretation that the two actions move in opposing directions.

**R1.3** *And in any case, the authors should discuss the potential impacts of these asymmetries on human behavior.*

- We agree that we did not adequately explain the possible effects of the asymmetries in the action-outcome mapping on the participants’ behavior. Accordingly, we have added the following text to the revised manuscript (lines 213-242):

The remaining three nuisance regressors were binary, each capturing an irregularity in the action-outcome contingencies. The reasoning behind these irregularities is further elaborated in the Methods section (section “action-outcome mapping”) and in S1 Appendix. Here, we discuss how these irregularities might influence response times, and how the three nuisance regressors aim to capture this influence. Firstly, four transitions per community were possible regardless of which choice the participant made (Fig. 1C, blue, “preferred”). Following a particular transition will elicit a prediction error, and larger prediction errors are hypothesized to yield slower responses for choices made in the subsequent state. Note that this is the basis for our prediction that participants should slow down following transitions between two different communities. Consider a participant who would behave completely randomly in the Museum task. In each room, they have a 50% chance of selecting either action, which yields a 50% chance of following either of two transitions as shown in Fig. 1B. A preferred transition could be elicited by both actions with a 50% chance, and would thus be experienced more often. Therefore, it is likely these preferred transitions yield lower prediction errors (i.e. they are predicted more strongly), yielding faster responses for the choices immediately following the transition. To capture this, we added a binary regressor that was 1 following a preferred transition, and 0 otherwise.

Secondly, some transitions were not possible in our design (Fig. 1C, red). Therefore, rooms with an outgoing red arrow as shown in Fig. 1C have only three unique possible outcomes in total (each action has two outcomes, but one outcome is shared between the two actions as a preferred transition). Notice that there is one room that does not have any removed or preferred transitions (labeled with “W” for “wide” in Fig. 1C). Because this is the only room that has 4 possible unique outcomes, participants might find it harder to decide on the most appropriate action in this room (as they have to consider more possible outcomes) predicting slower responses in this room. For this reason, we included a binary regressor that was 1 when participants occupied this wide room, and 0 otherwise.

Thirdly, transitions out of this wide room might be harder for participants to predict, given that each action has two unique outcomes as opposed to one unique outcome (with the other outcome shared between the two actions as a preferred transition in all other rooms). Outcomes that are harder to predict may yield larger prediction errors, and thus predict increased response times for the choices that immediately follow these transitions. For this reason, we included a binary regressor that was 1 following transitions out of this wide room, and 0 otherwise.

**Reviewer 2**

**R2.1** *There is a lot that I like about this manuscript. How people identify hierarchy in temporally extended tasks is an important problem in cognitive science. That the SR is used for this is a compelling hypothesis. The authors preregistered their analysis and mark where in their analysis they deviated from their preregistration. The model-fitting itself is very sophisticated, done in a bayesian manner with stan. Finally, using three different measures of processes (choice, response time, and free sort positioning) is clever.*

- We thank the reviewer for these encouraging words.

**R2.2** *The authors divide up their predictions into preregistered predictions and exploratory analysis. The preregistered predictions are that response times would reflect the SR and choices would reflect a strategy enabled by explicit hierarchical abstraction (EH). It is worth noting that this analysis alone would not really provide support that the SR, as revealed by response times, is connected to the explicit hierarchical abstraction as revealed by choice behavior. The key analysis which connects the response-time SR to choice-time hierarchical abstraction is an exploratory analysis, presented in Fig. 7. Here, it is shown that between-participant variability in the ability of the SR, fit to individual response times, to produce a marker of hierarchical abstaction (“Modularity”), explains between-participant variability in some makers of hierarchical abstraction in both choice and sorting behavior.*

- We agree that these are the main conclusions of our work and thank the reviewer for summarizing them so accurately. It is true that the correlation between SR modularity and measures of task performance is an exploratory analysis (i.e., was not preregistered), but we believe it is the correct hypothesis to test, as opposed to what we preregistered (relying on the participant-level model evidence in favor or the SR, as opposed to the modularity). Nevertheless, readers should be informed that this analysis was indeed not preregistered and draw their conclusions for themselves. When introducing this analysis, we emphasize its’ exploratory nature in lines 667-668 (“We conducted a series of exploratory analysis that …”). In response to this comment by the reviewer, we again emphasize this in the discussion section, where we added lines 821-824:

Further, the degree to which individual participants learned the community structure through the successor representation correlated with how effectively they applied a hierarchically abstract choice policy, although we note that this result is derived from an exploratory (i.e. not pre-registered) analysis.

**R2.3** *I think, in this analysis, the use of the SR “modularity” measure is somewhat obscuring a clear presentation of what precise aspects of variance in response times are relating to makers of hierarchical behavior in choice. My suspicion is that the key variability which SR “modularity” explains is not really specific to the SR itself, but rather mostly reflects variance in whether individuals tend to slow down after they transition from one cluster to another -- a clear marker of hierarchical abstraction in response time behavior -- though one which more models than just the SR could paramaterize.*

- We agree that other models than just the SR can also depend on discount factor-like parameter that places more or less emphasis on the community structure of the represented graph. In fact, the free energy – mental error model proposed by Lynn et al. (2020) has been shown to be mathematically related to the SR, making similar predictions specifically when considering ring-of-clique graphs as our museum task implements. In response to this comment by the reviewer, we have added the following text to the revised manuscript (lines 926-929):

Besides various implementations of the successor representation, our data could be explained by alternative sequence learning mechanisms, for example “free energy minimization” (29) or (cloned) hidden Markov models (91), in so far as these can implement a mechanism that is isomorphic to the discount factor of the successor representation.

**R2.4** *In this sense, I suspect the same variance would also be captured by the variance in the binary regressor in the EH model which tests whether participants changed clusters. Is this the case? If it is, it would not necessarily provide evidence that the SR is not the right model of response times, but it would suggest that the key variance in response times that relates to variance in evidence of hierarchy in choice is not specific to the SR model. More generally, it would permit a potentially simpler story that between participant variance in evidence for hierarchy in choices is related to between participant variance in evidence for hierarchy in response time (as opposed to parameterizing this variance with different models for choice and response time).*

- The upshot of this (and the previous) point is that response time variance related to task performance measures as parameterized by the modularity of the SR, might be fully explained by the slowing of response times when participants transition between communities. This is an important point, and a thorough investigation of this possibility relates to a different comment raised by this reviewer. In point R2.7, the reviewer suggests that we look for further evidence in favor of a successor representation model of response times, beyond the slowing of response times upon transitions between different communities. The text quoted there, as added in the revised manuscript in lines 501-524, shows how the successor representation model can capture meaningful variance in response times even when considering only transitions within communities, specifically the fact that participants are slower to respond for rooms that lie at the boundary between two communities, notably when excluding transitions between communities.
- A crucial follow-up question is whether the parameter estimation of the successor representation model of response times is uniquely informed by the response time slowing for transitions between communities, or if the response time variance displayed for transitions within communities essentially provides the same information. We investigated this by refitting all models when excluding transitions between communities from the likelihood. Doing so still shows significant preference for a successor representation model of response times, and this fit displays high correlation with the parameter estimation when including transitions between communities for all parameters including the discount factor. We added the following text to the revised S2 Appendix (section “excluding transitions between wings”):

Previous research has shown that participants tend to slow down their response times when transitions between stimuli across two different communities are experienced (5–7). The successor representation with a free discount factor parameter is especially suited to capture this pattern of data. It is interesting to see what would happen if we fit the different computational models to the same data, but exclude all trials following a between-community transition from the likelihood. If the successor representation remains the winning model, then this would indicate that it captures more variance than just that which can be described by the community structure of the environment, setting it further apart further from the explicit hierarchical model. In fact, when excluding all between-community transitions from the likelihood during model fitting, the successor representation is still the preferred model of response time data (BOR < 10^-11^, pxp = 1) (Fig S18A). This holds for all binary model comparisons between the successor representation and every other model (all BOR < 10^-4^, see Methods for multiple comparison correction. All pxp = 1).

Significance tests of all regressors lead to the same conclusion as in the main text, with inconclusive evidence for the influence of (log-transformed) number of trials on the current miniblock (“logsteps”) (M = -0.004, HDI95% = [-0.015, 0.007], ER- = 2.886), significant slowing for less (log-transformed) recent rooms (“logrecency”) (M = 0.029, HDI95% = [0.024, 0.035], ER+ = ∞), and significant slowing when participant changed their response key (“handswitch”) (M = 0.034, HDI95% = [0.013, 0.055], ER+ = 1094.890). Similarly, we find inconclusive evidence that participant slow down for the node with 2 unique transitions per response (“wide node”) (M = 0.004, HDI95% = [-0.004, 0.012], ER+ = 4.710) or in transitions that follow this node (“wide trans”) (M = 0.005, HDI95% = [-0.003, 0.013], ER+ = 6.416). We find significant slowing for responses following a “preferred” transition (M = 0.020, HDI95% = [0.011, 0.028], ER+ = ∞).

With respect to the regressors of theoretical interest (Fig S18B), we find significant slowing for transitions yielding higher state prediction error (“SPE”), indicating slowing when transitioning between two rooms with more different successor representations (e.g. “deep” rooms vs “boundary” rooms) (M = 0.011, HDI95% = [0.003, 0.020], ER+ = 304.344). We also find significant slowing for higher expected values (“EV”), indicating that participants respond slower for rooms closer to the current goal (M = 0.031, HDI95% = [0.023, 0.036], ER+ = ∞). We also find significant slowing for transitions with more negative reward prediction errors (“RPE”), consistent with post-error slowing when participants move away from the goal, or conversely, speeding when participants move toward the goal (M = -0.024, HDI95% = [-0.031, -0.017], ER- = ∞). Finally, we find no conclusive evidence that the regressor for conflict, conceptualized as the difference in expected value between the two different actions, influences response times (“conflict”) (M = 0.001, HDI95% = [-0.005, 0.007], ER+ = 1.472). The fact that all comparisons yield the same result is not surprising, given the high correlations between parameter estimates of the within-community model fit, compared to the parameter estimates of the full model fit (Fig S18C). Notably, the estimation of the discount factors is consistent whether between-community transitions are included or not (Fig S19). Since the modularity of the successor representation is a direct function of the value of the discount factor, this indicates that measures of modularity of the successor representation can be estimated robustly even without a quantification of response time slowing following transitions between communities. All the relevant model comparisons and parameter estimations are thus found to be significant in the same direction, regardless of including or excluding transitions between communities. We conclude that information about response time slowing between communities does not provide any information that cannot also be extracted from response time variance within communities.

- Further, response time slowing for transitions between communities should be directly related to the modularity of the successor representation. If the modularity is only a noisy derivative of the response time slowing between communities, then any correlation we compute between the modularity and a measure of task performance should vanish when we first account for the variance explained by response time slowing. However, if the modularity of the successor representation is informed by all transitions (including those within the same community), the modularity should be a more informative measure when correlated with measures of task performance, compared to response time slowing between communities. We directly tested this by replacing the originally reported simple linear regressions with hierarchical regression models, specifically controlling for response time slowing before testing the influence of modularity on measures of task performance. We show that response time slowing and modularity are indeed significantly related, but that modularity accounts for significant variance of various measures of task performance, above and beyond the measured response time slowing between communities. The significance level of the regression models is robust even to very strict multiple comparison correction (Holm-Bonferroni for 15 hypothesis tests, against an alpha of 0.05). We discuss this in the revised manuscript in the following text (lines 659-760):

Although there was strong evidence in favor of a successor representation model of response times across most individuals (see Fig. 3A), there were also large individual differences in the modularity of these successor representations. Note that the modularity is directly derived from the state prediction error metric. Hence, the degree of modularity of the successor representation for a specific participant could be completely driven by the relative slowing on between-community transitions (compared to within-community transitions). Additionally, the estimated modularity of the successor representation could be informed by response time variance within communities, such as the slowing for rooms at the boundaries of a community as reported in Fig. 5. This implies that modularity is in fact a more informative measure than response time slowing. We conducted a series of exploratory analyses that predicted independent task performance metrics based on the modularity of the response time-fitted successor representation models. To control for pure between-community response time slowing, we constructed hierarchical regression models where we first entered between-community response time slowing as the sole independent variable, and compare this against a second regression model including both response time slowing and modularity as independent variables. Between-community response time slowing was defined as the difference in mean response times between “outside” (green) and “between” (blue) transitions as indicated in Fig. 4A. The modularity of the successor representation is defined as the ratio of the state prediction error elicited by between-wing transitions over within-wing transitions (corrected for “preferred” transitions; see Methods). This tests whether the modularity of the successor representation provides information above and beyond the between-community response time slowing. In total, this exploratory analysis yielded 15 regression models (all individual panels of Fig. 8). When reporting the second-step model, we report the F and R2 statistics of the model itself, the slope of the newly added modularity regressor, and the ΔF statistic corresponding to the model comparison between the first- and second-step regression models. The reported p-value corresponds to this ΔF statistic. We corrected p-values for multiple comparisons across all first-step regression models and all comparisons with the second-step regression models (15 tests in total; see Methods for multiple comparison correction).

Our exploratory analysis confirmed a significant positive relationship between response time slowing and modularity (β = 0.001, F(1,117) = 7.472, R2 = 0.060, p = 0.044; Fig. 8A). Contrary to the posterior model probability of the successor representation (Fig. 7G), between-community response time slowing significantly predicted total accumulated reward bonus (β = 0.005, F(1,118) = 6.485, R2 = 0.052, p = 0.049; Fig. 8B, top). Including the modularity of the successor representation as a second independent variable leads to a significant improvement in model fit (β = 3.433, F(2,117) = 12.435, R2 = 0.175, ΔF(1,117) = 17.478, p < 0.001; Fig. 8B, bottom). Note that the total bonus payment depended solely on the participant reaching a new goal in the minimum number of steps possible. The time taken to generate these choices (the response time) was not related to reward, as participants could perform the task entirely at their own pace. Instead, this relationship is suggestive of a link between successor representation learning and task performance, where participants who are more sensitive to the community structure of the museum generate more efficient paths to the goal.

In accordance with the positive relationship between response time slowing and reward, we observed a significant positive relationship between response time slowing and the posterior means for the explicit hierarchical “rotation” regressor of the choice data (β = 0.003, F(1,117) = 14.281, R2 = 0.109, p = 0.002; Fig. 8C, top), indicating that participants whose response times were more affected by the community structure also reflected this community structure more strongly in their choice behavior. Including the modularity of the successor representation as a second independent variable leads to a significant improvement in model fit (β = 0.768, F(2,116) = 10.694, R2 = 0.156, ΔF(1,116) = 6.443, p = 0.037; Fig. 8C, bottom). In addition, the data shows a positive relationship between response time slowing and the degree of response-switching when participants left the goal wing (β = 0.003, F(1,117) = 14.642, R2 = 0.111, p = 0.002; Fig. 8D, top). Including the modularity of the successor representation as a second independent variable leads to a significant improvement in model fit (β = 0.995, F(2,116) = 11.810, R2 = 0.169, ΔF(1,116) = 8.090, p = 0.032; Fig. 8D, bottom). By contrast, no relationship is observed between response time slowing and response switching upon goal entry of the goal wing (β = 0.000, F(1,117) = 0.061, R2 = 0.001, p = 1; Fig. 8E, top). Including the modularity of the successor representation does not yield any improvement in model fit (β = 0.005, F(2,116) = 0.031, R2 = 0.001, ΔF(1,116) = 0.000, p = 0.979; Fig. 8E, bottom).

Although response times and choices reflect independent dimensions of the museum task, they are derived from the same behavioral unit (i.e. “the choice”). By contrast, the post-test free sorting task is completely independent from the behavior used to estimate modularity. Nevertheless, the data show a significant relationship between response time slowing and how close participants would sort paintings from adjacent rooms in the same wing (β = -0.002, F(1,116) = 18.926, R2 = 0.140, p < 0.001; Fig. 8F, top). Including the modularity of the successor representation as a second independent variable leads to a significant improvement in model fit (β = -0.991, F(2,115) = 26.401, R2 = 0.315, ΔF(1,115) = 29.264, p < 10^-5^; Fig. 8F, bottom). Interestingly, the data also showed a relationship between response time slowing and how far participants placed rooms that lie at the boundaries of the same wing, and are thus not connected (β = 0.001, F(1,116) = 16.069, R2 = 0.122, p < 0.001; Fig. 8G, top). Including the modularity of the successor representation as a second independent variable leads to a significant improvement in model fit (β = 0.377, F(2,115) = 26.851, R2 = 0.318, ΔF(1,115) = 33.177, p < 10^-5^; Fig. 8F, bottom). Higher values of both between-community slowing and successor representation modularity thus correspond to participants who correctly placed these rooms further apart, which in fact provides evidence against community structure and in favor of them accurately learning the true adjacencies. Similarly, the data showed a relationship between response time slowing and how closely participants would place two rooms that are connected between different wings (β = -0.002, F(1,116) = 17.548, R2 = 0.131, p < 0.001; Fig. 8H, top). Including the modularity of the successor representation as a second independent variable leads to a significant improvement in model fit (β = -0.872, F(2,115) = 28.219, R2 = 0.329, ΔF(1,115) = 33.912, p < 10^-6^; Fig. 8H, bottom). Higher values of both between-community slowing and successor representation modularity thus correspond to participants who correctly placed these rooms closer together, which is also in line with more accurate learning of true adjacencies. Collectively, these results indicate that participants who learned community structure using successor representations (as evidenced in response times), were better able to reconstruct the museum layout later from memory, while doing so with less bias towards reconstructing the underlying community structure.

**R2.5** *My other central methodological critique concerns the model comparison for response time data. The paper doesn’t explicitly consider alternatives to the SR for discovering hierarchy. In this sense, the explicit hierarchical (EH) model is a stand-in for potential alternative approaches.*

- While it is true that we do not actually consider any specific computational model for discovering hierarchical structure, it is important to realize that all these models (Correa et al. (2023), Stachenfeld et al. (2017), McNamee et al. (2016), Tomov et al. (2020)) assume a perfectly learned transition matrix of the underlying task. As shown by Correa et al. (2023) in simulations of these different models of hierarchical abstraction, they all converge on the same solution when the relevant feature for abstraction is a bottleneck transition between different communities. For this reason, we believe the EH model captures in a succinct way what a successful application of any of these models of hierarchical abstraction would predict, without relying on noisy estimation of the ground-truth transition matrix. In fact, the main point of our paper is to investigate a realistic model of the learning of such a transition matrix (the SR, which is not really a transition matrix) in the context of applying these models of hierarchical abstraction.

**R2.6** *Though, I felt like it was not entirely clear what aspects of the RT data led the SR to have an advantage over EH, and I worry that the advantage could potentially be due to superficial choices about what regressors are included for either model. Specifically, SR and EH don’t seem completely well-matched across types of regressors. As a model of response times, SR has 4 non-nuissance regressors: a) Current choice value, b) last RPE (difference in value between successive states), c) State prediction error (difference in SR between last two successive states), d) Current choice conflict - (difference between choice values for an action). EH in contrast only has 3 non-nuissance regressors: a) a Binary regressor indicating whether one is in the goal room or not (comparable to SR EV), b) a Binary regressor indicating whether last transition between wings or within wings (comparable to the SR SPE) c) binary regressor indicating whether one is in a room allowing for a transition (somewhat comparable to the SR’s conflict regressor, though potentially not exactly). This comparison shows that the EH model would be more directly comparable to the SR model if it added regressors comparable to the SR model (but without within-cluster variation) in the following dimensions: RPE and Conflict. Both of these can be derived by considering that the EH model effectively has 3 abstract states (one for each cluster in the task) and assigns a separate Reward for each of those abstract states. This abstract reward is 1 for the goal-containing cluster and 0 for the two non-goal clusters (deriving the ‘value’ of these abstract states is slightly more complicated as it needs to account for most ‘transitions’ leading back to the same state in which the action was taken, but we know that the value of the goal containing state is greater than the value of the two non-goal containing states). For the RPE component then, for EH this will be 0 following within-cluster transitions, something positive following a transition from a non-goal containing cluster to a goal-containing cluster, and something negative following a transition from a goal-containing cluster to a non-goal-containing cluster. For the conflict component, this will be 0 when both actions lead to no between-cluster-transisition, one value when one action leads to transitioning between a goal-containing-cluster and a non-goal-containing-cluster, and either a different value or 0 when one action leads to transitioning between two non-goal-containing-cluster. Overall, I’d say, since the conflict component doesn’t seem to significantly capture variance, adding the RPE component is more essential, but something more comparable to conflict would also be desirable so that the different model can be compared in their ability to predict the same type of quantities effect on response time, though they might differ in what they think that quantity is.*

- We thank the reviewer for this insightful comparison between the SR and EH models. We agree that the EH regressors proposed by the reviewer would make this model more appropriate for comparison with the SR. We fitted exactly this model as an additional control analysis. Comparing the SR with this more complex EH model increases the relative evidence for the SR (binary comparison BOR ≈ 0.001, as opposed to BOR ≈ 0.003), likely because of the inclusion of an additional regressor in the EH model (the RPE-like regressor) increasing the penalization for complexity of the EH model. For this reason, we stick to reporting the preregistered EH model in the main text and did not rerun any other control analyses with the more complex EH model. We report these results in S2 Appendix (section “a more equivalent explicit hierarchical model”) and quote the text here:

The explicit hierarchical model considered in the main text, consisted of three non-nuisance regressors, while the successor representation consisted of four. Where the successor representation modeled the expected value of the current room, the explicit hierarchical model encoded a binary value indicating whether the current room was part of the goal wing or not. Where the successor representation encoded a continuous state prediction error, the explicit hierarchical model encoded a binary value indicating whether a transition between two different communities was just made. These two variables provide a proper correspondence between the two models. However, where the successor representation encoded the conflict (implemented as absolute difference between expected value of the two choices), the explicit hierarchical model encoded a binary value indicating whether the current room allowed for a transition between two different communities. To make this correspond better to the actual conflict variable, we here consider a different binary value, that indicates whether the current room lies at the boundary allowing for a transition into or out of the goal wing. These rooms are assigned the highest conflict in the successor representation model. Most crucially, the successor representation encoded the reward prediction error, while the explicit hierarchical model did not have a regressor corresponding to this value. We included an additional variable that was coded as 1 for transitions into the goal community, as -1 for transitions out of the goal community, and as 0 otherwise. Even with this more equivalent definition of the explicit hierarchical model, the successor representation is the preferred model of response time data (BOR <10^-10^, pxp = 1) (Fig S20). This holds when considering a binary comparison between the explicit hierarchical and successor representation models (BOR = 0.001, pxp = 0.999).

**R2.7** *More generally, if the EH model is viewed as an RL model that just has 3 abstract states, there should really only be two potential sources of variance that the SR could capture that the EH doesn’t. The first would be variance in response times pertaining to different within-cluster events. EH can’t explain these because it treats the whole cluster as a single state. This would include RTs following within cluster-transitions (for various prediction error regressors) or RTs for conflict for actions that might transition to different within-cluster nodes (for conflict regressors). The second source would have to do with the policy-dependence of the SR - there might be some effect of previous trial’s goals on the SR which in turn would cause variance in response times for upcoming trials. I think showing that the SR captures variance on either of these dimensions would boost confidence that the SR indeed is capturing variance in response times that the EH model can’t (for a version of EH which might be more flexible than the specific one the current paper considers). As an example, this could potentially be done by limiting analysis to within-cluster transitions and showing that the SR SPE for example explains variance for these transitions alone.*

- We agree that more detailed predictions of the SR could be tested on our data. We could not think of an elegant test that would rely on the policy-dependence of the SR, and instead focussed on transitions within communities (of which models of hierarchical abstraction would not predict any variation in response times). Already in the original paper by Schapiro et al (2013) it was shown that nodes at the boundary of a community acquire representations that interpolate between the community they are a part of, and the community they are connected to. The successor representation replicates this representational structure, predicting that participants would slow down specifically in these boundary nodes even following transitions *within* communities. We report evidence for this in the revised manuscript in Fig. 5 and the following newly added text (lines 501-524):

To probe further how the successor representation might explain response times at a fine-grained (i.e. within-wing) level, we partitioned the trials as shown in Fig. 5A, indicating whether the current room lies at a boundary allowing for a transition to a different community and specifying whether this transition would correspond to entering the goal community (orange), leaving the goal community (yellow), or switching between non-goal communities (blue). These trials can be compared against trials within the goal community (pink) or outside of it (green). To ensure the variance in response times on these trials corresponds to knowledge of the within-community structure of the museum, we excluded all trials that followed a between-community transition. We conducted three paired t-tests to investigate sensitivity to the within-community structure of the museum (corrected for multiple comparisons; see Methods). With respect to rooms that are outside the goal community (Fig. 5B), we found a significant increase in response times in rooms at the boundary that allows for a transition between two non-goal communities (blue), compared to non-boundary rooms outside the goal community (green) (M_non-goal>outside_ = 6.921ms, SD_non-goal>outside_ = 32.292, t(119) = 2.348, p = 0.041). We also find a significant increase in response times for rooms at the boundary that allows for a transition “toward” the goal community (orange) compared to rooms at the boundary that allow for a transition between non-goal communities (blue) (M_toward>non-goal_ = 10.714ms, SD_toward>non-goal_ = 42.671, t(119) = 2.751, p = 0.021). Fig. 5C shows posterior predictions from the successor representation model. This reveals the model predicts a similar increase in response times for boundary-rooms that allow for transitions into the goal community (orange), but not for boundary-rooms that allow for transitions away from the goal community (blue; compared to non-boundary rooms outside the goal community, green). Inspection of the boundary and non-boundary rooms in the goal community (Fig. 5D) did not reveal statistically significant differences in response times (M_outof>within_ = 6.445ms, SD_outof>within_ = 79.123, t(119) = 0.892, p = 0.374), nor does the model predict any such differences (Fig. 5E).

- Additionally, we discuss how our state-action formulation might be misspecified to capture all the relevant response time variance in these boundary nodes. The following text was added to the revised manuscript (lines 910-925):

When inspecting posterior predictions of response times for various rooms within a single community (Fig. 5), we found that the state-action successor representation does not predict slowing in the boundary room that allows for a transition between two non-goal communities (blue). Because participants are prone to select the action that allows for rotation towards the goal community, they typically select the action allowing for a transition between communities when in the boundary room that allows for a transition toward the goal community (orange). Conversely, participants are not very likely to select the action allowing for a transition between communities when occupying the boundary room that allows for a transition between two non-goal communities (blue). This implies a smaller state prediction error for the latter rooms for the state-action successor representation as compared to the state-state successor representation, because outcomes that transition between communities are excluded in the former but not the latter case. As a consequence, specifically the state-action model incorrectly predicts less slowing for rooms at the boundary of two non-goal communities. Additionally, the boundary room that allows for a transition towards the goal community is assigned a substantially higher expected value than the other two types of rooms, further contributing to the model-predicted slower response times.

**R2.8** *It would be helpful if the paper could more clearly spell out the theory for how the proposed model of SR underlying abstraction leads to the type of between-participant RT - Choice relationships observed. If I understood, the broad explanation is that all participants learn the SR, but do so using different discount factors. All participants then apply computations to those SRs which enable discovery of abstractions which are used to guide choice, but not response times. However, those computations are only successful for SRs that are defined with high enough discount factors. Thus, only participants whose response times indicate high SR discount factors show evidence of hierarchy in choice. Is this the broad idea? If so, it might be nice to consider what applying secondary computations to SRs that are defined with lower discount factors would look like. Would this produce a recognizable choice behavior distinct from EH? If so, that might provide good additional evidence for this model.*

- We see this point as highly related to those raised in comments R2.9 and R2.10, since they address the underlying theory of how the SR learning would relate to hierarchical abstraction. We thank the reviewer for highlighting in detail the ways we could improve on the theoretical relationship between SR learning and hierarchical abstraction. We added lines 829-880 into the revised manuscript, addressing these issues jointly. Here, we deconstruct them into three separate points.
- We first note that the relevant models (Correa et al. (2023), Stachenfeld et al. (2017), McNamee et al. (2016), Tomov et al. (2020)) do have meaningful relationships with the SR, as further discussed in R2.10.
- Interestingly, the different models make different predictions of how the discount factor of the learned SR should relate to the effectiveness of the hierarchical abstraction. Graph-cut mechanisms as proposed by Stachenfeld et al. (2017) rely on the eigenvectors of the SR, which do not change with different discount factors. Approximations of the betweenness-centrality using randomized shortest paths (see R2.10) improve their sensitivity with lower discount factors (as then the SR better approximates a matrix of immediate transitions). Explicit divisions of the SR matrix into different clusters are most effective when the modularity is highest, which is the underlying mechanism of hierarchical abstractions proposed by McNamee et al. (2016) and Tomov et al. (2020). We thus conclude that our results are in line with secondary computations as proposed by McNamee et al. (2016) or Tomov et al. (2020). We added the following text to the revised manuscript discussing this (lines 829-859):

Several algorithms have been proposed that implement this secondary computation by taking an adjacency matrix as (part of) their input (13,15,16,23). This work generally assumes that the adjacency matrix has been accurately learned by the participants without considering how the specifics of the primary learning process could influence the abstraction process. Instead, we show that individual differences in learning a successor representation correlate directly with the efficacy of hierarchical abstraction. This finding is contrary to algorithms that rely on spectral graph theory wherein the relevant eigenvectors are constant over the entire range of possible discount factors (23), thus predicting no relationship between the estimated discount factors and the effectiveness of the hierarchical abstraction. Further, if abstractions rely on graph-theoretic measures of betweenness-centrality as described elsewhere (16), then the abstractions could be instantly computed from the successor representation as the “simple randomized shortest paths” betweenness, where the “fundamental matrix” corresponds directly to the successor representation (72, algorithm 1). However, the quality of this abstraction increases with smaller discount factors, which is the opposite of what we find. Additionally, subgoal identification through betweenness-centrality does not explicitly assign lower-level states to higher-level state abstractions, leaving open the question why people would apply a simplified general “rotational” policy to all states in one community.

By contrast, our findings are more consistent with a Bayesian model of hierarchical abstraction as proposed by (15). This model considers several factors for identifying efficient hierarchical representations, including dense connectivity within state abstractions, and sparse connectivity between them. When relying on a successor representation as input, as opposed to a ground-truth adjacency matrix, this density metric is by definition maximized when discount factors are most sharply tuned to capture the modularity of the environment. Similarly, (13, equation 7) propose an algorithm for computing the “entropic centrality” of different nodes. This computation can be performed directly by using the successor representation—which directly corresponds to the “fundamental tensor”—and substituting the degree of each node by the summed entries in the successor representation corresponding to that node. They observe that normative hierarchical abstractions often minimized differences in entropic centrality within state abstractions, and maximized this between state abstractions. This difference should be most sharply tuned in a successor representation when the discount factor emphasizes the modular structure most strongly, in line with our reported results.

**R2.9** *I was not convinced really by the explanation given in the discussion for why participants would adopt the EH strategy for choice, given that they use the SR for response times. The discussion argues that the EH strategy is chosen because it is less cognitively demanding and faster to execute. But why then would response times not also adopt and EH strategy? Relatedly, if SR values are computed for response times, why would it be cognitively demanding to then use those already computed values for choices as well?*

- While the original formulation of the successor representation showed how policies can be derived directly by multiplying the SR with a reward vector, later models have shown that sampling from the SR can have beneficial computational properties (Zhou, CY. et al., 2023) and predicts hippocampal replay observed during decision making tasks (Zhou, Z. et al., 2023). This might be especially important for making reliable decisions on a single-trial level. Meanwhile, the response time data displays high variance, and we only observe evidence for SR-like value computations by fitting the model to many trials across many participants. Hierarchical abstraction can make generative sampling from the SR more efficient. First, a high-level planner can sample sequences of abstract states that should be followed, then a lower-level planner can sample shorter sequences within these abstract states, instead of sampling sequences all the way to the goal state. Generalization of actions within abstract states can further alleviate this burden by omitting the requirement to run the lower-level planner at all. Our museum task affords the latter simplification. Our revised manuscript discusses this in lines 860-880:

Why would participants need to develop high-level (hierarchical) representations when the low-level successor representations already get the job done? Notably, both (13) and (15) explicitly assign lower-order states to higher-order state abstractions, and run separate planning algorithms at both levels. In each of these models, the lower-level planner should in principle be able to find policies with more fine-grained detail than following the simplified “rotational” policy. However, if we assume running the lower-level planner is computationally (72) (or cognitively, (6,56)) costly, agents might learn to omit running this planner, specifically when they can also learn that always executing the same action for every state in one state abstraction reliably yields good results (as in the Museum task). Although it has previously been argued that policies can be directly computed by multiplying the successor representation with a reward vector (24), which should not be computationally expensive, in more complex domains or for longer timescales, generative sampling may still be required to reliably asses the outcomes of the possible actions on a single trial based on a learned successor representation (73–75). Note that we only observe evidence for value computations based on a successor representation in the response times by fitting the model across many trials and many participants. Sampling from the successor representation would likely incur computational or cognitive costs (e.g. 75), which could be alleviated by hierarchical abstraction. We speculate that participants follow the rotational policy in the Museum task mainly for this reason, despite the fact that their response times reveal sensitivity to relatively detailed representations of the underlying task structure. That participants’ choice behavior can access such details is also illustrated by the significant decrease in the proportion of rotation choices when the rotation is in fact suboptimal (Fig. 2F-H).

**R2.10** *In the discussion, some cited papers are misdescribed. In one example, the discussion points to the following papers as having proposed a mechanism by which the SR can generate hierarchical representations (line 662): McNamee et al., 2016; Tomov et al., 2020; Correa et al., 2022; Stachenfeld et al., 2017. Of these papers, I think only the Stachenfeld paper actually proposes that the SR is used for hierarchical abstraction and proposes a computational process for this. McNamee proposes that abstraction is based on an information theory measure. Tomov proposes that it is based on bayesian inference given specific hierarchical graph priors. The Correa paper is about subgoal selection rather than state abstraction and doesn’t address the SR. I point this out only because I think it would be beneficial for this paper to somewhat more engage with previously made alternative proposals for what might guide state abstraction (and don’t say suggest that the SR is used for it). For example, the Tomov paper in particular presents analysis that explicitly argues against the SR as a model of abstraction. It would be worth it to consider the different conclusions reached there versus here.*

- It is true that only Stachenfeld et al. (2017) explicitly consider how the SR could be leveraged for hierarchical abstraction. However, the computations done by McNamee et al. (2016) implicitly rely on the SR as well (as a transformation of the ground-truth transition matrix, which they then term the “fundamental tensor”). Similarly, Correa et al. (2023) rely on a ground-truth transition matrix to algorithmically compute the betweenness-centrality of different nodes and show that participants are likely to select nodes with a higher betweenness-centrality as subgoals. Betweenness-centrality can be approximated by the SR when computing the “randomized shortest paths” betweenness-centrality (Kivimäki et al., 2016). The Bayesian model proposed by Tomov et al. (2020) could perform its inferences over the SR as well as over a ground-truth transition matrix. Where Tomov et al. (2020) argue their results are in disagreement with the SR, this specifically refers to computing a policy based on the SR directly, or after applying the graph-cut mechanisms as proposed by Stachenfeld et al. (2017). Their results should hold up if the SR was used as an input to their proposed Bayesian mechanism for hierarchical abstraction. All these models of hierarchical abstraction can thus be argued to have a meaningful relationship with the SR. We discuss this in the main text in lines 829-859 (see R2.8).

**Reviewer 3**

**R3.1** *Task: In the introduction, it is stated that the participant’s choice was probabilistically mapped to two of the four possible neighbouring rooms. But if I understood well, the two options were equiprobable. What was the motivation for these “balanced” action-outcome mappings? Are they required to adjudicate between the different models? Would the different models make similar predictions if the task structure would be more “conventional”, as in eg the original Schapiro study using community structure settings?*

- We are not entirely sure what the reviewer means with making the action-outcome mapping more “conventional” as in the original Schapiro study. The original Schapiro study, and many following studies that investigate the learning of community structure, provide sequences of stimuli drawn from a random walk on the graph shown in Fig. 1A. Because we wanted to investigate goal-directed behavior, we had to give participants some opportunity to influence the traversal of states in this graph. Crucially, we prevented against the participants explicitly selecting the path to take (e.g., select the next room out of 4 possible rooms, given the current room) because this would lead to the participants not experiencing any prediction errors, which are known to be large drivers of response times. In particular, robust findings of response time slowing following unpredicted transitions between communities, as observed for example in the study by Lynn et al. (2020), provided our main motivation to investigate this in a goal-directed setting. Our solution was to give participants the choice between two response keys, each leading to two different outcomes per room. S1 Appendix (lines “action-outcome mapping and node visit expectations”) elaborates exactly how we came to settle on the action-outcome mapping used in the main experiment; further, in response to Reviewer 1 (point R1.2), we added an additional section that discusses a possible alternative mapping (S1 Appendix section “a symmetric action-outcome mapping”).

**R3.2** *Models: All the models include regressors that appear fairly ad-hoc. For example, a regressor modeling the assumed increase in frustration for longer miniblocks, leading to speeded responses. Or a regressor modeling (inverse) “conflict” when there was a difference in “expected value” (with EV quantified how? Distance to goal?). Were all these extra regressors defined a priori, or a posteriori?*

- Notably, the regressors and the structure of our cognitive models were not ad-hoc, but rather were preregistered. We have now emphasized this in lines 188-190:

We preregistered the exact structure of the models, including the definition of all theoretically meaningful and all “nuisance” regressors (https://osf.io/n2jcz/; with exceptions marked and justified in S2 Appendix).

- Beyond our preregistration, we did add additional nuisance regressors to control for the asymmetry between “preferred” transitions and transitions from “wide” nodes. In response to point R1.3, we emphasized this in lines 213-242. S2 Appendix (section “original preregistration”) shows that our results are replicable even under the exact model with the exact regressors as preregistered. We also note that expected value is a quantity with a clear definition in the reinforcement learning literature (and in our methods section, lines 1101-1108, 1140-1141), and that participants are known to find it more difficult to select between options with similar expected values. To prevent the focus of our manuscript from becoming too broad, we mainly discuss literature on hierarchical reinforcement learning and the learning of community structure. We choose to not elaborate too much on the fundamental literature on reinforcement learning. Our hypothesis was that expected values and reward prediction errors would influence response times, as indicated by references included in lines 96-99 (e.g. Miletic et al. (2021)).

**R3.3** *Do the different models have different amount of explanatory regressors? If so, can that account for their ability to model the observed data? Is this taken into account in any way?*

- The included regressors for each model are described in the models section (lines 195-322). As pointed out in R2.6, the successor representation model in fact had one additional regressor compared to the alternative model-based and explicit-hierarchical models. As described under R2.6, we fitted a more elaborate implementation of the explicit hierarchical model (S2 Appendix section “a more equivalent explicit hierarchical model”) which actually slightly increases the relative evidence in favor of the successor representation. This is because of the Bayesian model comparison, which explicitly penalizes models in terms of their complexity (as described in the methods section, lines 1060-1067). We also note that our models share a fair amount of structure but are still recoverable in simulation, as detailed in S1 Appendix (section “parameter and model recovery”).

**R3.4** *Data: I would have liked to see some more “primary” data, showing eg the average amount of actions that participants needed to get to their goal state; and whether/how this changed over time, as they learnt the community structure. As it stands, Figure 2 immediately jumps to ‘model evidence’ for the different models.*

- We agree with the reviewer that a more convincing account of our data would include more direct presentation of the primary data, as opposed to relying solely on more opaque (but comprehensive) computational models and formal model comparisons.
- Firstly, we added lines 501-524 and Fig. 5 in response to R2.7. Here, primary data is shown and t-tests of raw response times are tested, to show participants are sensitive to the fact that nodes at the boundary of a community contain predictions of the adjacent community, increasing the dissimilarity with other rooms in the same community and thus predicting slower response times even following transitions within communities.
- Secondly, we further investigated the choice behaviour of participants. We added lines 395-419 and Fig. 2F-H investigating a specific situation where the explicit hierarchical choice model makes a prediction that differs from the model-based and successor representation models. Fig 2G shows primary data, indicating the proportion of “rotational” choices each participant has made for two specific rooms. In one of these rooms, the rotational choice was optimal (blue). In the other, it was not (orange). It can be seen that participants have a remarkable bias to select the rotational action, even when this is not optimal. This explains why the explicit hierarchical model is the best model of choice behavior. Interestingly, a significant difference in the proportion of rotational actions is observed, where participants were significantly less biased to pick the rotational action when this action was not optimal. This suggests some participants on some trials did realize that this specific room afforded a more optimal antirotational action. We describe these results in the revised manuscript in lines 395-419:

For each wing, given a goal in each other wing, there was always one room where the optimal choice according to a model-based (and converged successor representation) model would deviate from the explicit hierarchical model. This is because both the rotation and the antirotation can lead to the same desired outcome, but the rotation has a chance of transitioning to a room without connections towards the goal wing, whereas the antirotation does not (Fig. 2F). We set up an exploratory hierarchical Bayesian logistic regression to test whether participants are indeed more likely to follow the rotation when it is the optimal decision (blue, Fig. 2F) compared to when it is not (orange, Fig. 2F). Fig. 2G shows that in both of these rooms, regardless of which direction the rotation follows (<z> or <m>), participants select the correct rotation on most of the trials, but they do so less often when the antirotation offers a better alternative. A test of the intercept of the logistic regression confirms that participants are indeed overall biased towards selecting the rotation, as predicted by the explicit hierarchical model (M = 0.580, HDI95% = [0.489, 0.671], ER+ = ∞). In accordance with our earlier observations (Fig. 2C), participants are more likely to do so when the correct rotation is <m>, since they have a bias towards selecting this response (M = 0.621, HDI95% = [0.435, 0.809], ER+ = ∞; Fig. 2H, “rotation”). Crucially, when participants occupy the room where the rotation is the optimal direction (blue, Fig. 2F), they are more likely to select the rotation, compared to when the antirotation is in fact optimal (orange, Fig. 2F) (M = 0.097, HDI95% = [0.048, 0.145], ER+ = 9999.000; Fig. 2H, “node type”). This effect is numerically, although not significantly, attenuated when the correct rotation is <m> (M = -0.057, HDI95% = [-0.156, 0.040], ER- = 7.061; Fig. 2H, “interaction”). Overall, this confirms that participants’ choice behavior is mostly explained by an explicit hierarchical model in agreement with our model comparison, as evidenced by the large influence of the rotation even when this action is not in fact optimal. However, small influences of more precise (model-based or successor-like) knowledge is evident at the group level, indicating that the best model might allow for a mixture of hierarchical abstraction and fine-grained knowledge (cf. 43).

- Furthermore, we note that the originally submitted manuscript contained primary data, which is still included in Fig. 2C (plotting the proportion of choices following the correct rotation), Fig. 2E (plotting the proportion of changes of direction following entering the goal community and leaving the goal community), Fig. 4C (plotting mean response times following the transition types indicated in Fig. 4A), and Fig. 6A (plotting the distances between the paintings as placed in a free sorting task).

**R3.5** *Hypotheses: I found it difficult to understand how the SR model differed from the “optimal model-based decision maker”. It is stated that the SR model holds a multi-step predictive model, whereas the model-based model holds a one-step predictive model. But why is that the case? In MB decision-making, one would also calculate the value of all the future possible actions and use those to derive the optimal action policy? In the cited paper by Momennijad, it was nicely explained how an experimental setting was created that could distinguish between MB and SR. Here, I could not understand how the models would lead to different predictions.*

- We agree that a more intuitive account of our data would emphasize explicitly how the computational models differ from each other and additionally test for these differences using primary data (as discussed in R3.4). We do note that S1 Appendix (under “computational models”) contains a full exposition of the differences in (choice) behaviour predicted by the different computational models.
- Firstly, as described in R3.4 and elaborated in R2.7, we added lines 501-524 and Fig. 5. This addresses a specific prediction of the successor representation w.r.t. response times within communities. Specifically, participants should be slower for rooms at the boundary of two communities, since their representation should interpolate between the two communities and thus be markedly different from rooms deeply nested in a single community. This response time slowing should be present even when considering transitions within communities (as opposed to transitions between communities, which predict even further slowing of response times). Neither the model-based nor the explicit hierarchical cognitive models have an explanation for this pattern in the data. This analysis hopefully provides readers with an intuition for why the successor representation is the winning model for response times.
- Secondly, we provide a more detailed investigation of the choice data (see R3.4). We added Fig. 2F-H and lines 395-419 specifically investigating a situation where the explicit hierarchical (winning) model differs from the model-based and successor representation models. This is because of our action-outcome mapping including preferred transitions. When the current goal lies outside of the current community, two rooms can be identified in the current community that both allow for a transition in the direction of the current goal. One of these actions corresponds to the “rotational” action, and is thus the action that would allow for the actual transition into the goal community. The other action is the “antirotational” action, and thus does not allow for this transition. Interestingly, in one room (the orange room in Fig. 2F) this rotational action either leads in the direction of the goal community, or to a room that does not allow for a transition in the direction of the goal community (a room with a removed edge, red cross in Fig. 2F). Crucially, the antirotational action also allows for a transition in the direction of the goal community, but also for a transition to an adjacent room that still has an edge in the direction of the goal community. Therefore, it is optimal to select the antirotational action when occupying the orange colored room in Fig. 2F. Nevertheless, the data show that participants are significantly biased to pick the rotational action even in this room, although intriguingly they do so less often than when the rotational action is in fact the optimal choice (see R3.4).
- In conclusion, we presented two new tests. Lines 395-419 discuss how the (winning) explicit hierarchical model explains most variance in the choices and specifically deviates from the SR and the model-based predictions, though there is evidence of SR and/or model-based knowledge in the choices as well. Lines 501-524 discuss how participants slow down their response times for rooms at the boundary of a community, and specifically more when this allows for a transition into the goal community. This pattern of predictions is uniquely explained by the (winning) SR model, in contrast to the explicit hierarchical and/or model-based model.
